# Adaptation and Validation of a Scale for Measuring the Curriculum-Based Professional Learning Community in Early Childhood Education in China

**DOI:** 10.3389/fpsyg.2022.909842

**Published:** 2022-07-22

**Authors:** Qian Peng, Lijia Liu, Limin Zhang, Yaping Yue

**Affiliations:** ^1^Department of Early Childhood Education, School of Education, South China Normal University, Guangzhou, China; ^2^Department of Early Childhood Education, School of Education, Guangzhou University, Guangzhou, China; ^3^Faculty of Education, Henan University, Kaifeng, China

**Keywords:** teacher's self-efficacy, early childhood education, Chinese context, curriculum-based professional learning community, model testing

## Abstract

Teachers' professional learning community, as an effective path to promote teachers' professional development and elevate teaching quality, has been widely used in school contexts. In preschools, the practice of teachers' professional learning community mainly focuses on the curriculum in early childhood education. The revision and adaptation of the scale of the professional learning community for preschool teachers in the Chinese cultural context are of great significance for understanding the current situation of the professional learning community for preschool teachers and improving the quality of collaboration within the community. Teachers' Professional Learning Community scale was revised into the Curriculum-Based Professional Learning Community scale according to the characteristics of the curriculum in early childhood education in the Chinese context. Based on the data from a sample of 2,823 teachers, the study conducted an item analysis and exploratory factor analysis (EFA) on participants from sample A (*N* = 1,410) and confirmatory factor analyses (CFAs) on participants from sample B (*N* = 1, 413). Short-form Teacher Self-Efficacy scale was used as the criteria-related validity instrument. Sample A and sample B were used to explore the relationship between various dimensions of teachers' professional learning community and teachers' teaching efficacy. The results showed that instead of the five-factor structure of the original PLC scale, the Chinese version of the CBPLC scale consists of four factors: Shared Sense of Purpose, Collective Focus on Children Learning and Development, Collaborative and Reflective Activity, and Deprivatized Practice. The revised scale has high reliability and validity and can be used as an effective tool to measure the curriculum-based professional learning community of preschool teachers in China. The results of CFA indicated that the four-factor CFA model fit the data well, and the CBPLC significantly and positively predicted teachers' self-efficacy including instructional strategies, students' engagement, and classroom management.

## Introduction

Recently, adult learning is more and more viewed as a process of full participation in communities of practice that requires situated learning or on-the-job training (Lave and Wenger, 1991). Over the past decades, “work-based learning” has become a focal issue in the field of research on teacher professionalization (Tynjälä, 2013). The way of teachers' professional development has changed from a top-down perspective with the assumption that teachers are lacking the sufficient skills and knowledge and need to be educated by external experts, to a bottom-up perspective which is teacher-driven, purposeful for teaching practice, and directed toward improving student learning (Bergmark, 2020). Numerous studies in teacher education believe that teacher-centered collaborative learning can bring both teachers' professional development and students' achievement growth (Chong and Kong, 2012; Akiba and Liang, 2016; Moy et al., 2021). Collaborative learning can afford an opportunity for teachers to establish wellconnected networks through which they share and rethink their educational practice and pedagogical beliefs, as a result co-construct knowledge through social interaction (Chan and Pang, 2006).

Professional learning communities (PLCs) have been seen as an important tool to enhance teachers' collaborative learning. The literature on PLCs shows that PLCs can facilitate teacher development (Stoll et al., 2006; Huijboom et al., 2021), improving teachers' knowledge and classroom practice (Andrews and Lewis, 2007) and students' achievement (Buysse et al., 2010; Bergmark, 2020; Wasik and Hindman, 2020). The construction of PLCs has become a centerpiece of many proposals for restructuring schools and supporting teachers' classroom practice and professional development (Newmann and Wehlage, 1995; Louis et al., 1996; Louis and Marks, 1998; Garet et al., 2001). As for preschool teachers, PLCs can promote preschool teachers' teaching experience, improving teachers' ability of critical thinking and reflection, expanding the overall experience of individual teachers, and maintaining a harmonious interpersonal relationship on the campus through constantly instructional problem-centered communication with colleagues (Liu, 2018). Further, with the increasingly complex roles of preschool teachers, it is necessary to improve teachers' professional development through PLCs. Although the positives of PLCs are often discussed, there is limited information about preschool teachers' experiences in a PLC (Damjanovic and Blank, 2021).

With the development of PLCs, the curriculum-based professional learning community (CBPLC) has received increasing attention recently. Darling-Hammond et al. (2017) pointed out that effective teachers' professional development should focus on curriculum and classroom. Voogt et al. (2011) concluded that the practice of collaborative curriculum design can facilitate teacher learning processes. CBPLC guides a series of focused, small-group sessions, cooperative learning, peer cooperation, and reflective teaching discussion to solve curriculum and instruction problems, allowing teachers to teach according to the needs of the students. Teachers' subject knowledge and pedagogical content knowledge (PCK) can be developed in CBPLC through rehearsing lessons and focusing on common concerns (Polly et al., 2016; Short and Hirsh, 2020). CBPLC can close the gap between adult's views and child's perspectives, challenging teachers' beliefs, overcoming teachers' teaching obstacles, and enhancing their teaching confidence and teaching satisfaction (Polly et al., 2016; Darling-Hammond et al., 2017; Short and Hirsh, 2020; You et al., 2021).

Research on PLCs in the ECE context is rare but growing. And it has been encouraged to conduct more PLC research in the Chinese cultural contexts (Hairon et al., 2017; Yin et al., 2019). Since the “new curriculum reform” launched in 2001, curriculum decentralization has made preschool-based curriculum development as the basic tendency of curriculum reform in early childhood education in China, which means higher requirements for the professionalization of preschool teachers. Therefore, the professional standards for preschool teachers (Trial) issued by the Ministry of Education of China in 2012 proposed that preschool teachers should “have the spirit of teamwork, actively carry out cooperation and communication, learn together, constantly reflect and forge ahead…. Cooperate and exchange with colleagues, share experience and resources and develop together.” PLCs have become a basic way to promote the construction of teams of preschool teachers and the improvement in instruction quality. Some Chinese scholars refer to the teaching and research groups (TRGs), lesson preparation groups, and research groups of schools in the stage of basic education in China as PLCs or potential PLCs (Sargent and Hannum, 2009; Qiao et al., 2018).

Researchers from European and American countries have designed scales to measure PLCs (Louis and Marks, 1998; Olivier et al., 2009). Due to cultural differences, PLCs in Chinese preschools are different from those in the Western. Therefore, PLCs measurement tools derived from Western situations are not necessarily suitable for the Chinese context. We hope to revise the measurement tools of CBPLC suitable for Chinese preschools. Studies have identified a positive relationship between teachers' self-efficacy and teachers' participation in a PLC (Stegall, 2011; Porter, 2014). CBPLC may increase the possibility of this kind of relationship with teachers' self-efficacy. The study chose the relationship between CBPLC and teacher self-efficacy for model testing.

### Teachers' Professional Learning Community

The notion of PLCs proposed by DuFour and Eaker (2009) refers to a group of organized teachers sharing and reflecting their practice and beliefs in a collaborative, collegial, practice-oriented, and, teaching- and learning-oriented, teacher-oriented way to enhance teachers' professional development (Hord, 1997; Stoll and Louis, 2007; Darling-Hammond et al., 2009). As teachers collaborate with colleagues on effective instructional practices in the work-embedded context, with the help of experts outside, PLCs became renowned as the solution to teachers' personalized learning and struggling alone, and a valid means for realizing collaborative decision making, improving teachers' satisfaction, and facilitating students' achievement (DuFour and Eaker, 2009; Hord and Sommers, 2009). Of course, it is necessary to make a distinction between the romanticized notion of PLCs and the facts which exist in PLCs' practice with tension at times, competition, and even a lack of enthusiasm in participating (Damjanovic and Blank, 2021).

Different researchers seem to ascribe different characteristics to a PLC, and recently, people began to realize that the form of PLC does not necessarily bring ideal results and high-quality PLC needs conditions to support. The description of PLCs' features presents two research paradigms. One paradigm focuses on describing the characteristics of the operational process of PLCs such as collaboration, reflection, and giving and receiving feedback (Louis et al., 1996; Huijboom et al., 2021). The other paradigm focuses on the conditions required for a well-functioning PLC such as supportive and shared leadership (Hord, 1997; Hord and Sommers, 2009), deprivatization of practice, and its results such as collective learning and shared values (Hord, 1997; Louis and Marks, 1998; Hord and Sommers, 2009). Alongside these characteristics, professional learning integrated in the daily practices of specific social communities is also highlighted (Tynjälä, 2013).

### The Teacher Professional Learning Community Scale

Professional Learning Community Assessment (PLCA) was used to assess everyday classroom and school-level practices according to the conceptualization of the PLC dimensions and related attributes (Huffman and Hipp, 2003) which is revised as Professional Learning Community Assessment—Revised (PLCA-R), which include six factors: shared and supportive leadership, shared values and vision; collective learning and application; shared personal practice; supportive conditions—relationships; and supportive conditions—structures. It can be seen that the scale from Louis and Marks (1998) has always occupied the mainstream. Based on the hypothesis that a schoolwide professional community defined as an element of school organizational culture can help teachers become “better teachers,” Louis and Marks (1998) investigated the relationship between a PLC and classroom organization and, subsequently, student learning. A five-factor model is proposed by Louis et al. (1996) and Louis and Marks (1998), which includes five components: shared value, collective focus on student learning, collaborative activity, reflective dialogue, and deprivatized practice. Fundamental to the school professional learning community are shared values and expectations which teachers affirm about children, instruction, teachers' roles, the relationship between teachers and students, and so on. A collective focus on student learning is central to the school professional community by leading teachers to provide appropriate teaching and learning opportunities to promote students' development. To improve their skills for effective instruction, teachers share expertise through collaborative professional development activities such as peer coaching, teamed teaching, structured classroom observations, and reflective dialogue about teaching and learning in the professional community, which increases teachers' sense of affiliation with each other and with the school. This conceptualization that emphasizes collaboration and organizational supporting for teacher learning has been validated in the Chinese context (Yin and Zheng, 2018; Huang et al., 2020).

### Professional Learning Communities (PLCs) as Learning Environments for Teachers

As an example of a PD model, PLCs may create a supportive learning environment for the teacher through a reflective, collaborative, and teacher-centered approach nested in teachers' everyday experiences in the classroom. However, PLCs are also such an environment as teachers' engagement in the PLC around the social construct through identification and negotiability. Whether PLCs are seen as a worthwhile endeavor as a viable means of professional development depends on whether the PLC can bring teachers a sense of belonging (Damjanovic and Blank, 2021) and is work-embedded (Darling-Hammond et al., 2017; Damjanovic and Blank, 2021). Ideal PLCs should incorporate elements of effective professional development by providing chances for active, collaborative, and reflective learning for teachers, focusing on students' learning and supporting student achievement in the end (Darling-Hammond et al., 2017).

Strong professional communities have a positive effect on the job satisfaction of preschool teachers because PLCs create mutual learning chances for them, allowing them to share responsibility for students, permitting them to develop the best strategy for teaching through social interaction, and designing, implementing, and improving curriculum with collective efforts (Stearns et al., 2014). According to Huijboom et al. (2021), the concept of PLCs is a joint learning of individual and collective learning and creating collective knowledge. Important conditions that PLCs facilitate teachers' professional development include the followings: opportunity to collaborative learning, support from school leaders and experts, authorized collective autonomy, time, space and sources, teachers' teaching needs met, and organizing reflective instruction (Darling-Hammond et al., 2017; Huijboom et al., 2021).

### CBPLC and Preschool Teachers' Professional Development

Based on the theoretical framework of social learning, studies emphasized a professional development process based on collaboration, specific situations, and job-embedded model (Penuel et al., 2007; Bergmark, 2020; Short and Hirsh, 2020). Effective teachers' professional development should provide teachers coaching and other supports to cater to teachers' needs (Zhang et al., 2019, 2020), facilitating teachers' problem-based involving-in-learning (Short and Hirsh, 2020). PLCs allow teachers to combine professional development skills and knowledge for instructional planning, diagnosis of student learning, and action research for re-teaching (DuFour et al., 2008). The integrated nature of curriculum, assessment, and instruction is the most important and fundamental factors in effective teaching (Marzano, 2003; Roach et al., 2008). Recently, significant efforts to develop high-quality curriculum materials are aligned with educational reform and teachers' professional development (Penuel et al., 2007). The reason for this is that the Utopia of reform will not be transformed into daily classroom practice without the marriage of a new curriculum and teacher learning (Ball and Cohen, 1996). PLCs have been proved to be an effective way to empower teachers (Stoll et al., 2006; Huijboom et al., 2021). In particular, curriculum-based professional learning placed the focus on a curriculum which rooted in teachers' ongoing, active experiences and allowed teachers to experience instruction as their students will. That stands in contrast to traditional teacher training, which typically relays a static mass of information that teachers selectively apply to existing practice. The research showed that curriculum-based professional learning can prompt teachers to change their instructional practices, expand their content knowledge, and challenge their beliefs (Polly et al., 2016). Among curriculum-based professional learning, the Lesson Study approach developed the preschool teachers' content knowledge as they designed, taught, and reflected upon early number of lessons (Leavy and Hourigan, 2017). Compared with traditional teachers' professional development, CBPLC calls for several major shifts: focuses on the curriculum goals to promote teacher professional learning with instructional materials with specific teaching strategies, grinders class collectively instead of fighting alone, possesses a lot of opportunities for curriculum-focused coaching, reflecting and feedback, experiences inquiry-based learning, and models the sense-making strategies teachers will apply to students (Short and Hirsh, 2020).

CBPLC can change teachers' beliefs in their teaching and young children's learning, such as adopting learner-centered pedagogy, improving teachers' PCK and practice ability, and improving young children's achievement finally (Polly et al., 2013, 2016; Wasik and Hindman, 2020).

### Professional Learning Community and Teachers' Self-Efficacy

In the past few decades, the study of self-efficacy from the perspective of cognition or social cognition has become a hot spot. Teachers' self-efficacy (TSE), theoretically coming from the concept of self-efficacy, is defined as the confidence teachers hold about their capabilities in a specific teaching situation to bring about expected teaching outcomes (Bandura, 1982; Tschannen-Moran et al., 1998; Klassen et al., 2011; Locke and Johnston, 2016). According to Locke and Johnston (2016), teacher self-efficacy has developed into a two-dimensional construct: the self-perception of teaching competence and the sense of task difficulty. The measurement of TSE is mainly from the following three dimensions: Efficacy for Instructional Strategies (TSE-IS), Efficacy for Classroom Management (TSE-CM), and Efficacy for Student Engagement (TSE-SE) (Tschannen-Moran and Hoy, 2001).

PLCs increase teachers' self-efficacy (Reeves, 2010), and several researchers attributed the improvement in teachers' self-efficacy to the cooperative attribute and the continuous improvement in PLCs (Porter, 2014; Zonoubi et al., 2017). Cooperations in PLCs create opportunities for teachers to experience sharing, discussion on effective teaching strategies, and acceptance of suggestions and feedback from experts and their colleagues (Wahlstrom and Louis, 2008; Zonoubi et al., 2017). The model of continuous improvement existing in PLCs not only improves teachers' pedagogical knowledge and teaching ability, but also facilitates teachers' self-efficacy (Zonoubi et al., 2017). The four sources of efficacy information mentioned by Bandura (1997) also conceptually support the proposition that PLCs serve as a space for the development of teachers' self-efficacy (Zonoubi et al., 2017). Several pieces of research show that learning opportunities teachers consider available in the working situation have a significant positive prediction on teachers' self-efficacy (Lakshmanan et al., 2011; Stegall, 2011; Porter, 2014).

### The Current Study

The current academic community has reached a basic consensus on the definition and characteristics of PLC, and the practice of PLC has been studied using both quantitative and qualitative research methods. Improving teachers' instructional practices plays a key role in the improvement in early childhood education and the healthy physical and mental development of young children (Keung et al., 2020). However, research on PLCs in the Chinese context has focused on the primary and secondary school levels, with less research on the current state of PLCs in preschools. In addition to this, there is a lack of measurement of teacher PLCs that has been tested for good reliability and validity based on the context of early childhood education.

Specifically, the two objectives of this study are as follows:

1. To explore issues related to the measurement of CBPLCs and to revise the original scale to adapt to the Chinese cultural context for early childhood education.
2. To verify the model proposed in this study by exploring the relationship between CBPLCs and preschool teachers' self-efficacy.

## Methods

### Stage 1: Revising the Chinese Version of the Curriculum-Based Professional Learning Community Scale

#### Participants

Based on the characteristics of the Chinese kindergarten management structure, a snowball approach was used to collect the questionnaires. The researcher forwarded the web link of the questionnaire to the principals of kindergartens across China, who in turn distributed it to their kindergarten teachers for completion.

The total number of questionnaires returned was 2,947, of which 2,823 were valid, with an effective rate of 95% (2,823/2,947). Participants were arbitrarily divided into two subsamples utilizing the random split function in SPSS V26. Sample A (*N* = 1,410) was used for conducting EFA in stage 1, and sample B (*N* = 1,413) was used to test factor structure and concurrent validity in stage 2. The demographic information distribution of the respondents of sample A and sample B is given in Table 1. To confirm that sample A and sample B have the same validity in the next data analysis procedure, we use the chi-square difference test to examine whether the two samples were significantly different in these demographic characteristics. The chi-square difference results showed that there was no significant difference between sample A and sample B in terms of gender, age, professional title, position, budgeted post, the residence of preschool, and types of preschool (all *p* values > 0.05; gender [${x^{2}}_{\left(1\right)}$ = 0.04, *p* = 0.84]; age [${x^{2}}_{\left(5\right)}$ = 2.09, *p* = 0.83]; professional title [${x^{2}}_{\left(4\right)}$ = 1.84, *p* = 0.76]; position [${x^{2}}_{\left(5\right)}$ = 6.32, *p* = 0.27]; budgeted post [${x^{2}}_{\left(4\right)}$ = 4.225, *p* = 0.37]; residence of preschool [${x^{2}}_{\left(2\right)}$ = 1.01, *p* = 0.60]; types of preschool [${x^{2}}_{\left(2\right)}$ = 0.38, *p* = 0.82]).

**Table 1:** Demographics of participants (*N* = 2,823).

		**Sample A** **(*****N*** **= 1,410)**		**Sample B** **(*****N*** **= 1,413)**	
**Demographic characteristic**	**Code in SPSS**	***N***	**%**	***N***	**%**
**Gender**					
Female	1	1,391	98.7	1,397	98.9%
Male	2	19	1.3	16	1.1%
**Age**					
<22	1	273	19.4	262	18.5
23–25	2	289	20.5	314	22.2
26–30	3	271	19.2	268	19.0
31–35	4	237	16.8	247	17.5
36–40	5	176	12.5	165	11.7
>40	6	164	11.6	157	11.1
**Professional Title**					
No title	1	1,004	71.2	1,015	71.8
Third title	2	88	6.2	95	6.7
Second title	3	207	14.7	198	14.0
First title	4	101	7.2	91	6.4
Senior title	5	10	0.7	14	1.0
**Position**					
Assistant teacher	1	601	42.6	622	41.9
Head teacher	2	524	37.1	501	33.7
Grade/teaching and research group leader	3	178	12.6	184	12.3
Administrative positions such as deputy director	4	118	8.3	115	7.8
Other position	5	64	4.5	63	4.2
***Bianzhi*** **(budgeted post)**					
Owned	1	82	5.8	97	6.9
Non-owned	2	1,328	94.2	1,316	93.1
**Residence of preschool**					
City	1	746	52.9	759	53.7
Township	2	551	39.1	530	37.5
Rural	3	113	8.0	124	8.8
**Types of preschool**					
Public	1	643	45.6	661	46.8
Private inclusiveness	2	560	39.7	547	38.7
Private	3	207	14.7	205	14.5

#### Measure

This study translated the Professional Learning Community scale developed by Louis and Marks (1998) and revised it in order to fit the context of early childhood education and the characteristics of the “curriculum in early childhood education.” The original PLC scale is an English questionnaire that has been applied in a Chinese context (Yin et al., 2019). First, the original scale was translated into Chinese by a master's student in preschool education and a PhD student with 3 years of overseas study experience and was revised according to the actual situation of the early childhood curriculum.

Second, an associate professor of English education was invited to back-translate the scale from Chinese to English. Comparing the different parts of the two English translations, revise them and then translate them into Chinese, we analyzed and evaluated the two Chinese translations, and 10 preschool in-service teachers working in preschools were invited to put forward suggestions on revising the ambiguous sentences in the scale or those inconsistent with Chinese expression habits. Finally, with the approval of the revised scale by two associate professors and senior experts who have been engaged in early childhood education research for many years, a Chinese version of the Curriculum-Based Professional Learning Community (CBPLC) scale was formed.

The original scale consists of five dimensions: Shared Sense of Purpose (3 items), Collaborative Activity (6 items), Focus on Student Learning (4 items), Deprivatized Practice (4 items), and Reflective Dialogue (2 items). Item 7, “I make a conscious effort to coordinate the content of my courses with other teachers” in the “CA” dimension, does not correspond to the actual situation of the preschools in the Chinese context. Therefore, we remove this item and replace it with “Teachers plan and work together to search for measures to meet diverse student needs.”

#### Data Analytic Strategy

A series of exploratory factor analyses (principal component analysis) were performed using IBM SPSS V26 to explore the latent structure of the CBPLC scale which is adapted from the original PLC scale.

### Stage 2: Confirmatory Factor Analysis and Validity of the CBPLC Scale

#### Participants

Sample B (*N* = 1,413) was utilized for the CFA and to supply initial evidence of validity by testing anticipated relationships with theoretically similar constructs (teacher self-efficacy).

#### Measures

##### Demographic Questionnaire

In the demographic questionnaire, basic information about the teachers was collected, including teachers' age, gender, position, professional title, *bianzhi* (budgeted post), types of preschools, and residence of preschools.

##### Curriculum-Based Professional Learning Community

We measured the curriculum-based professional learning community using the 19-item scale validated in stage 1. The original scale consists of five subscales: shared sense of purpose, collective focus on student learning, collaborative activity, deprivatized practice, and reflective dialogue. Cronbach's alpha was 0.972. Each item was appraised on a six-point Likert-type scale, extending from 1 = *strongly disagree* to 6 = *strongly agree*.

##### Teacher Self-Efficacy

The teacher self-efficacy scale (12 items, TSE-short), which was developed by Tschannen-Moran and Hoy (2001), was used in this study. The scale consists of three dimensions: efficacy for instructional strategies, efficacy for classroom management, and efficacy for student engagement. Each item was appraised on a six-point Likert-type scale, extending from 1 = *strongly disagree* to 6 = *strongly agree*. Teacher self-efficacy was reported to be significantly associated with teacher professional community (Zheng et al., 2018b). Tschannen-Moran and Hoy (2001) provided high reliability estimates, higher than 0.90. Zheng et al. (2018a) reported that the reliability of the Chinese version of the scale was estimated to be higher than 0.86. In our research, Cronbach's alpha was 0.95.

#### Data Analytic Strategy

To examine the construct validity of the CBPLC scale, we first conducted confirmatory factor analysis (CFA) using maximum likelihood estimations to access the model fit which was run to test five models to choose the best structure of CBPLC. Model fit indices χ^2^, χ^2^/*df* ratio, Tucker–Lewis index (TLI), comparative fit index (CFI), standardized root mean square residual (SRMR), and root mean square error of approximation (RMSEA) are used to access the goodness of fit of the models. According to Hu and Bentler (1999), when the model fit index is χ^2^ (*p* < 0.05), χ^2^/*df* ≤ 3, CFI ≥ 0.90, TLI ≥ 0.90, and RMSEA and SRMR < 0.80, the model is considered to be a good fit. In addition to this, we used a chi-square difference test to rule out the possibility that the five models were not significantly different from each other. Mplus 8.0 was used for data analysis.

## Results

### Exploratory Factor Analysis (Sample A)

Sample A (*N* = 1,410) was used for EFA. Before conducting exploratory factor analysis, we utilized the Kaiser–Meyer–Olkin test and Bartlett's test to prove the appropriateness of the collected data for factor analysis. The Kaiser–Meyer–Olkin measure of sample adequacy was 0.974, whereas the Bartlett's test of sphericity was significant (*p* < 0.001), showing that the data were suitable for an EFA, as suggested by Tabachnick et al. (2007).

Based on the factor analysis using varimax rotation, after limiting the number of extracted factors, we obtained four structures, including a two-factor structure, a three-factor structure, a four-factor structure, and a five-factor structure.

In the two-factor structure, factor 1 consisted of 16 items (eigenvalue = 13.225, variance explained = 69.607%) and factor 2 consisted of 3 items (eigenvalue = 5.856, variance explained = 75.472%).

In the three-factor structure, factor 1 consisted of 14 items (eigenvalue = 13.225, variance explained = 69.607%), factor 2 consisted of 3 items (eigenvalue = 5.856, variance explained = 75.472%), and factor 3 consisted of 2 items (eigenvalue = 0.644, variance explained = 78.859%).

In the four-factor structure, factor 1 consisted of 9 items (eigenvalue = 13.225, variance explained = 69.607%), factor 2 consisted of 5 items (eigenvalue = 5.856, variance explained = 75.472%), factor 3 consisted of 3 items (eigenvalue =0.644, variance explained = 78.859%), and factor 4 consisted of 2 items (eigenvalue = 0.514, variance explained = 81.565%).

In the five-factor structure, factor 1 consisted of 8 items (eigenvalue = 13.225, variance explained = 69.607%), factor 2 consisted of 5 items (eigenvalue = 5.856, variance explained = 75.472%), factor 3 consisted of 3 items (eigenvalue = 0.644, variance explained = 78.859%), factor 4 consisted of 2 items (eigenvalue = 0.514, variance explained = 81.565%), and factor 5 consisted of 1 item (eigenvalue = 0.417, variance explained = 83.578%). In this structure, the last factor consists of only one item. As suggested, we removed the five-factor structure.

Table 2 shows the clustering of factor loads for each item and the correlation between each factor and the corresponding factor. According to Comrey and Lee (2013), a factor load greater than 0.4 was used to consider the variable as significant.

**Table 2:** Results of the exploratory factor analysis on the CBPLC in sample A (*N* = 1,410).

**Items**	**Factor loading**								
	**Two-factor**		**Three-factor**			**Four-factor**			
	**F1**	**F2**	**F1**	**F2**	**F3**	**F1**	**F2**	**F3**	**F4**
CBPLC1		0.670		0.552				0.829	
CBPLC2		0.851			0.835			0.881	
CBPLC3		0.891			0.886			0.531	
CBPLC4	0.674		0.684			0.704			
CBPLC5	0.566		0.707			0.677			
CBPLC6	0.802		0.714			0.549			
CBPLC7	0.812		0.711			0.704			
CBPLC8	0.827		0.746			0.691			
CBPLC9	0.794		0.733				0.667		
CBPLC10	0.821		0.727				0.681		
CBPLC11	0.815			0.747					0.785
CBPLC12	0.822			0.775					0.712
CBPLC13	0.805		0.734				0.599		
CBPLC14	0.664		0.640			0.746			
CBPLC15	0.815		0.816				0.754		
CBPLC16	0.828		0.842				0.795		
CBPLC17	0.840		0.827			0.717			
CBPLC18	0.774		0.731			0.730			
CBPLC19	0.812		0.758			0.714			
Eigenvalues	13.225	5.856			0.644				0.514
% of variance explained	69.607%	75.472%			78.859%				81.565%

### Confirmatory Factor Analysis (Sample B)

We conducted a series of CFAs for sample B (*N* = 1,413). Five models were tested and compared.

*Model 1: One-factor model. All items of the CBPLC loaded on a single latent factor (a unidimensional one-factor model)*.

*Model 2: Two-factor model. The model structure is derived from EFA*.

*Model 3: Three-factor model. The model structure is derived from EFA*.

*Model 4: Four-factor model. The model structure is derived from EFA*.

*Model 5: Five-factor model. The model structure is derived from the original study by Louis and Marks (**1998**)*.

Table 3 shows the fit indices and results of chi-square difference test of each model, and it can be seen that model 4 has a better fit than the other models.

**Table 3:** Confirmatory factor analyses in sample B (*N* = 1,413).

**Model**	**χ^2^**	***df***	**χ^2^/** ***df***	**SRMR**	**TLI**	**CFI**	**RMSEA**	**Comparison**	**χ^2^ diff test**
1	3,452.028	152	22.711	0.033	0.871	0.885	0.124	–	
2	2,660.040	151	17.616	0.027	0.901	0.913	0.108	2 vs 1	791.98(<0.001)
3	2,184.790	149	14.663	0.018	0.919	0.929	0.098	3 vs 2	475.25(<0.001)
4	1,718.827	146	11.773	0.020	0.936	0.945	0.080	4 vs 3	465.96(<0.001)
5	2,353.112	142	16.571	0.027	0.908	0.923	0.105	5 vs 1	1,098.9(<0.001)

*Model 1, one-factor model; model 2, two-factor model; model 3, three-factor model; model 4, four-factor model; model 5, five-factor model. N = 1,413; χ^2^= chi-square statistic; CFI, comparative fit index; TLI, Tucker–Lewis index; RMSEA, root mean square error of approximation; SRMR, standardized root mean square residual; χ^2^Diff test, chi-square difference test, ANOVA test*.

#### One-Factor Model

This model has a poor fit and can be considered unsuitable to be adopted as the structure of CBPLC (χ^2^/*df* = 22.711, CFI = 0.885, TLI = 0.871, SRMR = 0.033, and RMSEA = 0.17).

#### Two-Factor Model

Most of the fit indices of this model are considered to be of an acceptable degree, but the value of RMSEA is on the high side (χ^2^/*df* = 17.616, CFI = 0.913, TLI = 0.901, SRMR = 0.027, and RMSEA = 0.108). The two-factor model exhibited a better fit than the one-factor model, as reflected by a significant chi-square difference (Δχ^2^ = 791.98, *p* < 0.001).

#### Three-Factor Model

This model has a better fit than the two-factor model, but there are still some unsatisfactory fit indices (χ^2^/*df* = 14.663, CFI = 0.929, TLI = 0.919, SRMR = 0.018, and RMSEA = 0.098). The three-factor model exhibited a better fit than the two-factor model, as reflected by a significant chi-square difference (Δχ^2^ = 475.25, *p* < 0.001).

#### Four-Factor Model

The fit indices of this model are the best among all models, and all reach the desired interval (χ^2^/*df* = 11.773, CFI = 0.945, TLI = 0.936, SRMR = 0.020, and RMSEA = 0.080). The four-factor model exhibited a better fit than the three-factor model, as reflected by a significant chi-square difference (Δχ^2^ = 465.96, *p* < 0.001).

#### Five-Factor Model

The dimensions of this model are divided according to the dimensions of the original PLC scale. However, the model fit results showed that the model fit index of this model was poor and did not apply to the adapted CBPLC scale (χ^2^/*df* = 16.571, CFI = 0.923, TLI = 0.908, SRMR = 0.027, and RMSEA = 0.105). This model is a nested model of model 1 and has a significant chi-square difference from model 1 (Δχ^2^ = 1098.9, *p* < 0.001).

### Concurrent Validity (Sample B)

This step evaluates the concurrent validity of the CBPLC by examining its correlation with teacher self-efficacy. Teacher self-efficacy was measured by 12 items developed by Tschannen-Moran and Hoy (2001). The Chinese version of this scale has been validated in China (Cheung, 2008; Liu and Hallinger, 2018). The correlations between each dimension of CBPLC and teacher self-efficacy were examined. Table 4 reports the results of the correlation coefficients of the study variables. As expected, CBPLC was found to be positively associated with teacher self-efficacy (r = [0.299,0.890], *p* < 0.001). It was found that the correlation between the CRA and DP dimensions was high (β = 0.89) and the possibility of variable covariance needed to be excluded. We used the tolerance statistic and the variance inflation factor (VIF) to demonstrate the absence of multicollinearity among the four dimensions. In statistics, it is generally accepted that tolerance values less than 0.1 point to the presence of multicollinearity and VIF values greater than 10 indicate multicollinearity. After calculation, the value of each dimensional tolerance is greater than 0.1 and the value of VIF is less than 10. There was no evidence of multicollinearity in this study. The results of the test are given in Table 5. PLCs were generally able to positively influence teacher self-efficacy, with the amount of explanation for both reaching extremely significant levels.

**Table 4:** Correlation coefficients of CBPLC with teacher self-efficacy (*N* = 1,413).

	**TSE-IS**	**TSE-CM**	**TSE-SE**	**CRA**	**DP**	**SSOP**	**CFCLD**	**CBPLC**	**TSE**
TSE-IS	1								
TSE-CM	0.736***	1							
TSE-SE	0.745***	0.825***	1						
CRA	0.485***	0.404***	0.441***	1					
DP	0.498***	0.432***	0.469***	0.890***	1				
SSOP	0.368***	0.299***	0.325***	0.714***	0.643***	1			
CFCLD	0.423***	0.352***	0.383***	0.774***	0.710***	0.674***	1		
CBPLC	0.501***	0.421***	0.458***	0.975***	0.930***	0.810***	0.833***	1	
TSE	0.896***	0.930***	0.932***	0.481***	0.506***	0.358***	0.419***	0.499***	1

* p < 0.05, ^**^p < 0.01, ^***^p < 0.001. TSE-IS, efficacy for instructional strategies; TSE-CM, efficacy for classroom management. TSE-SE, efficacy for student engagement; CRA, Collaborative and Reflective Activity; DP, Deprivatized Practice; SSOP, Shared Sense of Purpose; CFCLD, Collective Focus on Children Learning and Development. *, ** statistical table.

**Table 5:** Tolerance and variance inflation factor of CBPLC four dimensions.

**Variance**	**Tolerance**	**Variance inflation factor (VIF)**
CRA	0.159	6.300
DP	0.210	4.759
SSOP	0.462	2.164
CFCLD	0.366	2.735

**Figure 1:**
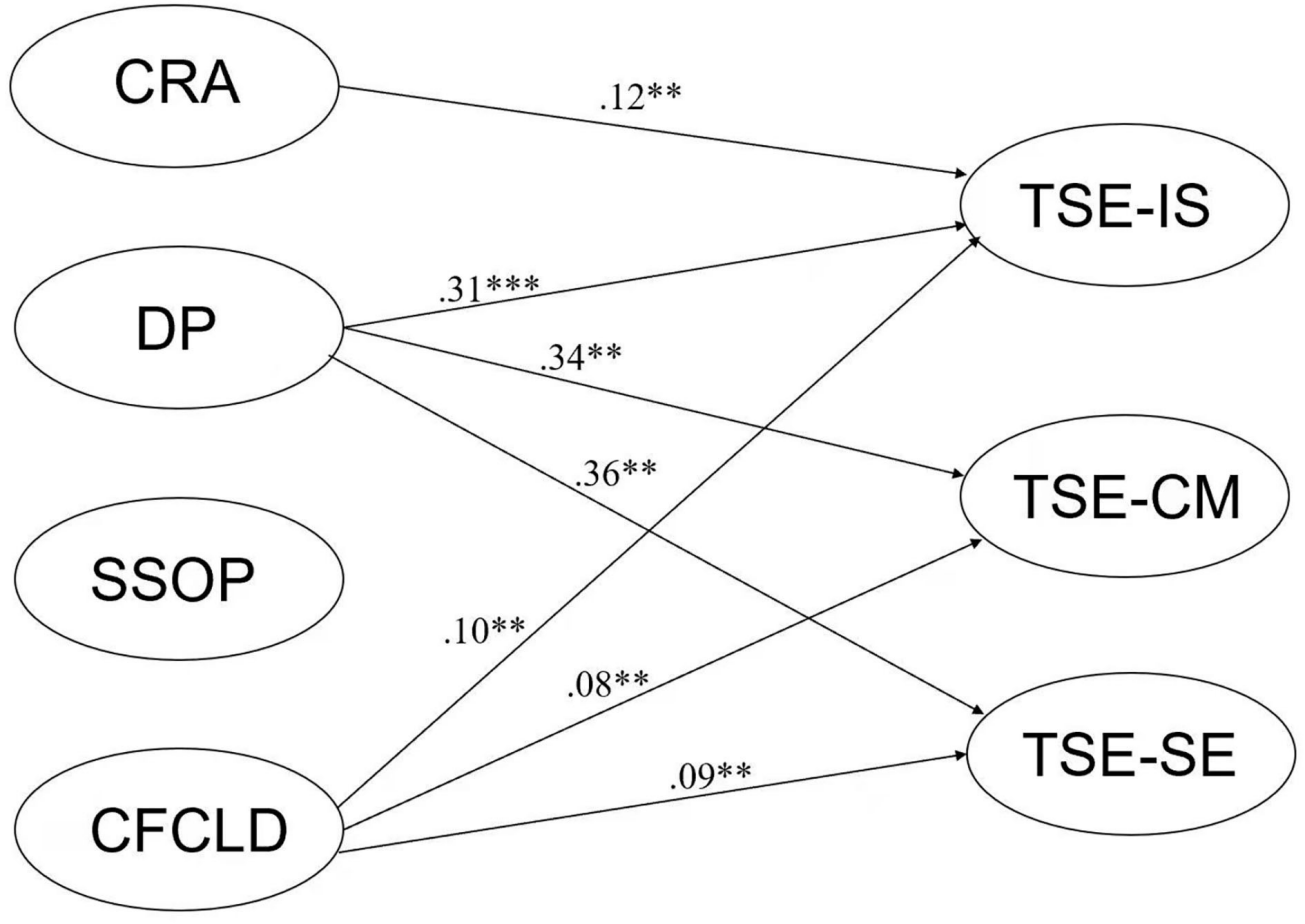
SEM of CBPLC and TSE. ***p < 0.001, **p < 0.01. TSE-IS, efficacy for instructional strategies; TSE-CM, efficacy for classroom management. TSE-SE, efficacy for student engagement; CRA, collaborative and reflective activity; DP, deprivatized practice; SSOP, shared sense of purpose; CFCLD, collective focus on children learning and development.

Second, the author constructed a SEM of the relationship between the four elements of CBPLC and teacher self-efficacy. The results of the model are shown in Figure 1. The model has a good fit (χ^2^ = 3567.88, *df* = 413, *p* < 0.001, RMSEA = 0.052, CFI = 0.965, TLI = 0.96). The graph only shows the paths that reached the significant level (*p* < 0.05). As shown in Figure 1, the four elements of CBPLC explained the three dimensions of teaching efficacy (R^2^) at a significant level (*p* < 0.01). Specifically, DP positively predicted all three dimensions of teacher self-efficacy across the four dimensions of professional community, and CFCLD had a significant positive effect on teaching strategy efficacy (β = 0.18, *p* < 0.01). Notably, SSOP had no effect on teaching efficacy in the model, while CRA had a negative and significant effect on teachers' classroom management efficacy (β = −0.13, *p* < 0.01).

## Discussion

The research described in this article aims to revise and validate the Curriculum-Based Professional Learning Community (CBPLC) scale as a measurement tool in the Chinese ECE context. This is the first measurement tool that integrates the kindergarten curriculum with a professional learning community for teachers.

In stage 1, the original PLC scale was translated by using the classic back-translation method. An item that did not correspond to the actual situation of kindergartens was deleted, and an item related to the collaborative activity of teachers was added.

The EFA tested in stage 1 showed that the dimensions of the revised scale had some adjustments from the original scale. This is because the original scale was administered to teachers from primary and secondary schools, and the form of professional learning community for these groups differs significantly from that of preschool teachers. The nature of the ECE context would seem to be conducive to collaborative practices because teachers usually work in the same physical space and interact frequently throughout the whole working day (Thornton and Cherrington, 2018).

The CFA was conducted to confirm which structure of the CBPLC obtained from stage 1 has the best model fit. Five models were examined in stage 2: a one-factor model, a two-factor model, a three-factor model, a four-factor model, and a five-factor model. The four constructs model was the best fit for the data.

The first dimension of the CBPLC scale contains nine items (items 4, 5, 6, 7, 8, 14, 17, 18, and 19) which include all the items from the Collaborative Activity and Reflective Dialogue dimensions of the original PLC scale. These two dimensions describe the learning, reflective, and dialogic activities that preschool teachers engage in collectively. Therefore, the first dimension of the CBPLC scale was named “Collaborative and Reflective Activity.” The second dimension of the CBPLC scale consisted of five items (items 9, 10, 13, 15, and 16). Most of the questions in this dimension were derived from the “Deprivatized Practice” dimension of the original scale, so the naming of this dimension remains unchanged. The third dimension of the CBPLC consists of three items (items 1, 2, and 3). This dimension is exactly the same as the “Shared Sense of Purpose” dimension of the original scale, so the naming of this dimension remains unchanged. The fourth dimension of the CBPLC scale contains two items (items 11 and 12). These two items come from the “Collective Focus on Student Learning” in the original PLC scale, so the name of this dimension remains unchanged. In summary, the four dimensions of CBPLC are as follows: (a) Collaborative and Reflective Activity (CRA), 9 items; (b) Deprivatized Practice (DP), 5 items; (c) Shared Sense of Purpose (SSOP), 3 items; and (d) Collective Focus on Children Learning and Development (CFCLD), 2 items.

Why does CBPLC in ECE in China present a four-factor model instead of a five-factor model? This may be due to the different cultures and practice in the preschool's PLCs between China and Western countries. In China, due to the cultural orientation of collectivism, preschools' PLCs tend to integrate such factors as cooperating in curriculum construction and collective teaching research through collective reflection and dialogue mainly to improve the whole teaching quality. Therefore, in practice, it shows the integration of collaborative activity, reflective dialogue, and shared practice of the original table. China's Ministry of Education issued Several Opinions on Improving and Strengthening Teaching Research in 2001, calling for the establishment of a “school-based teaching research system.” In February 2012, China's Ministry of Education issued the professional standards for preschool teachers (Trial), which further emphasized that preschools should carry out preschool-based research and promote teachers' professional development (Ministry of Education of the People's Republic of China, 2012). Preschool-based teaching research for all teachers mainly takes collective teaching research, such as lesson study, class listening and evaluation, collective lesson preparation, and topic discussion. With the decentralization of curriculum power and the strengthening of the curriculum leadership consciousness of the principle of preschool, the construction of preschool-based curriculum and class-based curriculum has become an important content of preschool-based teaching research. Preschool-based teaching research makes comprehensive use of individual reflection and collective reflection, with a focal point of collective reflection, that is, through collective teaching research and with the help of individual reflection to promote collective reflection, to solve some common problems of preschool teachers and promote the improvement in the overall curriculum and teaching quality of preschools. Demand-oriented and problem-centered approach has become the current popular trend of PLCs in China's preschools, that is, the community emphasizes teaching research activities based on preschool teachers and children's development and needs to solve problems. This is true for rural public preschools, especially for urban preschools.

The results show that the interpretation amount (R^2^) of the four elements of CBPLC on the three dimensions of teacher self-efficacy reached a significant level (*p* < 0.01) and there was an extremely significant positive correlation between CBPLC and all dimensions of teacher self-efficacy. PLCs increase teachers' self-efficacy (Reeves, 2010) because of their collaborative nature (Porter, 2014) and reflective nature, school collective atmosphere (Meristo and Eisenschmidt, 2014; Aldridge and Fraser, 2015). Teachers attributed teacher self-efficacy growth to the characteristics of the PLCs as described above just because they provide activities such as collaborative discussions, peer observations, improving teachers' knowledge and skills for teaching, classroom management, and teacher–student interaction (Zonoubi et al., 2017). The above interpretation also applies to China.

Among the four dimensions of PLCs, DP significantly and positively predicts the three dimensions of TSE. According to the scale this study is revising, DP describes that preschool teachers participate in teaching research activities regularly with the help of preschool principals, scholars outside and peers through listening to and evaluating lessons each other, and reviewing and discussing children's activities and performance in class collectively. PLCs in early childhood education in China are the cluster professional orientation which includes shared mental models, contributing to the effectiveness of PLCs (Huijboom et al., 2021). DP improves teachers' collaboration in case of well-connected teacher social networks and teachers' self-efficacy as a result (Slavit et al., 2011; Voelkel, 2011; Moolenaar et al., 2012).

The reason why Collective Focus on Children Learning and Development (CFCLD) positively predicts teachers' three dimensions of self-efficacy is that CFCLD means that teachers always pay attention to children's learning and development, especially in the pedagogy and curriculum, rather than a certain achievement or performance as the activity result. The guiding outline for early childhood education (Trial) issued in 2001 puts forward educational concepts such as lifelong learning, happy childhood life, and respect for children's personality and rights. Since the Ministry of Education issued the Guide for the Learning and Development of Children Aged 3–6 in October 2012, early childhood education research and training institutions across the country have been actively studying, interpreting, and implementing it. Teachers' learning in the PLC focuses on teaching strategies, class management, teacher–student interaction, and students' participating in learning. As a result, teachers' knowledge, ability, and belief in student learning are improved, which enhances teachers' self-efficacy.

Collaborative and Reflective Activity (CRA) only significantly predicts teachers' efficacy of instructional strategy (IE), which has no relationship with the other two dimensions of teacher self-efficacy. The reasons lay in CRA focusing on teachers' joint commitment to the curriculum construction of preschools and teachers' teaching. And the content of teachers' collective learning does not involve or rarely involves specific matters such as class management and students' involvement.

In the model, SSOP (shared sense of purpose) does not affect teachers' self-efficacy. This conclusion is not completely consistent with the research conclusion of Zheng et al. (2018a). Zheng et al. (2018a) believes that in terms of the research sample, the shared sense of purpose has a significant positive impact on TSE-IS and TSE-SE. Of course, the establishment condition of these relationships is that teachers have a high sense of identity with the value of the school and the planning objectives of the school (short-term and long-term objectives), and the development vision and curriculum concept of the school is formed after the joint discussion of teachers. The fact is that the popular authoritarian management mode makes the development vision and curriculum concept and cooperation of Chinese Preschools more top-down as shown in a recent empirical study of teachers in East Asia (Chen et al., 2018).

## Conclusion

Revising and adapting the scale of preschool teachers' professional learning community to the Chinese cultural context is important for understanding the current situation of preschool teachers' professional learning community and improving the quality of cooperation within the community. This study first used the back-translation method to translate the PLC scale and adapted it to the curriculum context in early childhood education, resulting in the CBPLC scale. The four-factor model of the CBPLC scale was validated as the most appropriate structure based on the analysis of the data returned from a large-scale questionnaire. The four factors were named as (1) Shared Sense of Purpose, (2) Collective Focus on Children Learning and Development, (3) Collaborative and Reflective Activity, and (4) Deprivatized Practice. Finally, the relationship that CBPLCs can positively predict teacher self-efficacy was also validated.

## Data Availability Statement

The original contributions presented in the study are included in the article/supplementary files, further inquiries can be directed to the corresponding author.

## Ethics Statement

Ethical review and approval was not required for the study on human participants in accordance with the local legislation and institutional requirements. Written informed consent from the participants was not required to participate in this study in accordance with the national legislation and the institutional requirements.

## Author Contributions

QP drafted the manuscript. LZ designed the research and revised the manuscript. LL and YY collected and extracted the data for analysis. All authors have approved the final version of this article.

## Funding

This work was supported by the Project of Humanities and Social Sciences of Ministry of Education of the People's Republic of China [21YJC880092].

## Conflict of Interest

The authors declare that the research was conducted in the absence of any commercial or financial relationships that could be construed as a potential conflict of interest.

## Publisher's Note

All claims expressed in this article are solely those of the authors and do not necessarily represent those of their affiliated organizations, or those of the publisher, the editors and the reviewers. Any product that may be evaluated in this article, or claim that may be made by its manufacturer, is not guaranteed or endorsed by the publisher.
